# The Study of Plasticized Amorphous Biopolymer Blend Electrolytes Based on Polyvinyl Alcohol (PVA): Chitosan with High Ion Conductivity for Energy Storage Electrical Double-Layer Capacitors (EDLC) Device Application

**DOI:** 10.3390/polym12091938

**Published:** 2020-08-27

**Authors:** Shujahadeen B. Aziz, Jihad M. Hadi, Elham M. A. Dannoun, Rebar T. Abdulwahid, Salah R. Saeed, Ayub Shahab Marf, Wrya O. Karim, Mohd F.Z. Kadir

**Affiliations:** 1 Hameed Majid Advanced Polymeric Materials Research Laboratory, Department of Physics, College of Science, University of Sulaimani, Qlyasan Street, Kurdistan Regional Government, Sulaimani 46001, Iraq; 2 Department of Civil Engineering, College of Engineering, Komar University of Science and Technology, Kurdistan Regional Government, Sulaimani 46001, Iraq; rebar.abdulwahid@univsul.edu.iq (R.T.A.); ayub.shahab@gmail.com (A.S.M.); 3 College of Engineering, Tishk International University, Kurdistan Regional Government, Sulaimani 46001, Iraq; jihad.chemist@gmail.com; 4 Department of Medical Laboratory of Science, College of Health Sciences, University of Human Development, Kurdistan Regional Government, Sulaimani 46001, Iraq; 5 Associate Director of General Science Department, Woman Campus, Prince Sultan University, P. O. Box 66833, Riyadh 11586, Saudi Arabia; elhamdannoun1977@gmail.com; 6 Department of Physics, College of Education, Old Campus, University of Sulaimani, Kurdistan Regional Government, Sulaimani 46001, Iraq; 7 Charmo Research Center, Charmo University, Peshawa Street, Chamchamal, Kurdistan Region, Sulaimani 46001, Iraq; salah.saeed@charmouniversity.org; 8 Department of Chemistry, College of Science, University of Sulaimani, Qlyasan Street, Kurdistan Regional Government-Iraq, Sulaimani 46001, Iraq; wrya.karim@univsul.edu.iq; 9 Centre for Foundation Studies in Science, University of Malaya, Kuala Lumpur 50603, Malaysia; mfzkadir@um.edu.my

**Keywords:** plasticized polymer electrolyte, polymer blend, ammonium salt, XRD analysis, impedance study, energy storage device

## Abstract

In this study, plasticized films of polyvinyl alcohol (PVA): chitosan (CS) based electrolyte impregnated with ammonium thiocyanate (NH_4_SCN) were successfully prepared using a solution-casting technique. The structural features of the electrolyte films were investigated through the X-ray diffraction (XRD) pattern. The enrichment of the amorphous phase with increasing glycerol concentration was confirmed by observing broad humps. The electrical impedance spectroscopy (EIS) portrays the improvement of ionic conductivity from 10^−5^ S/cm to 10^−3^ S/cm upon the addition of plasticizer. The electrolytes incorporated with 28 wt.% and 42 wt.% of glycerol were observed to be mainly ionic conductor as the ionic transference number measurement (TNM) was found to be 0.97 and 0.989, respectively. The linear sweep voltammetry (LSV) investigation indicates that the maximum conducting sample is stable up to 2 V. An electrolyte with the highest conductivity was used to make an energy storage electrical double-layer capacitor (EDLC) device. The cyclic voltammetry (CV) plot depicts no distinguishable peaks in the polarization curve, which means no redox reaction has occurred at the electrode/electrolyte interface. The fabricated EDLC displays the initial specific capacitance, equivalent series resistance, energy density, and power density of 35.5 F/g, 65 Ω, 4.9 Wh/kg, and 399 W/kg, respectively.

## 1. Introduction

The development of electrochemical devices such as batteries, and electrical double-layer capacitors (EDLCs) has attracted the attention of many researchers, because of their wide applications in portable devices [1]. The most favorable electrochemical storage devices are EDLCs with higher energy properties [2]. EDLC is an energy storage device that supplies electricity via producing an electrical double layer consisting of an adsorbed layer of anions and cations at the electrolyte/the electrodes interfaces. However, traditional batteries deliver lower power and store adequate energy than the traditional capacitors [1,3]. The EDLC with relatively high conductive polymer as electrolytes and electrode separators, might be a potential substitute for other types of charge storage devices [4]. Recently, EDLC has attracted much attention because of its unique properties like durability, speedy charge-discharge rate, higher energy density, reversibility, and improvement in safety, which make it a good candidate for a wide range of applications [5]. In the fabrication of EDLCs, the electrode of activated carbons (ACs) plays a vital role as an activated material owing to the excellent chemical and physical characteristics for instance; large temperature stability, high specific surface area, cheap and good conductivity that can be prepared from the variety of forerunners with the activated agents through heat treatment. Correspondingly, coal is the most popular source of activated carbon manufacturing due to its abundance, cost-effective, and high content of carbon that makes it preferable [6,7,8]. Good mechanical property and high ionic conductivity are promising for the EDLCs preparation. Besides, liquid electrolyte exhibits high ionic conductivity, nonetheless, there is a leakage problem and corrosion while gel and solid-state electrolytes overcome these drawbacks, and they are considerably preferred in EDLCs [9].

Solid polymer electrolyte (SPE) as a key material for various solid-state electrochemical devices have been reported [10,11]. SPE can be prepared by dissolving inorganic salts in a polar polymer chain, and the salts provide anions and cations that make the polymers conductive ionically [12,13]. It is well established that biodegradable polymers are more desirable over non-degradable polymers in the polymer-based electrolytes, because of their biocompatibility and renewability. Among the biodegradable polymers, polyvinyl alcohol (PVA) and chitosan (CS) are two of the utmost valuable candidates as environmentally friendly materials in the preparation of SPEs [14,15]. Previous studies have shown that, CS can be successfully employed in the energy storage devices [16,17]. The PVA polymer exhibits excellent outstanding characteristics including hydrophilicity, high chemical stability, the large capacitance of charge storage, safety, and affordability [18]. Additionally, the presence of a hydroxyl group (OH) in the PVA backbone structure can be considered as the source of the hydrogen bond and promoting the formation of polymer electrolytes [19]. On the other hand, chitosan is mostly investigated and it has a variety of performances in different fields because of the enrichment of functional groups (i.e., NH_2_, OH) in its backbone structure [20]. The amino group in the CS structure is able to act as the electron donner and strongly coordinates with the inorganic salts which make it enable to generate the proton-conducting SPEs [21].

Blending of polymers is a practical methodology undertaken to increase the room temperature ionic conductivity and reduce the crystallinity of polymers. PVA as a semi-crystalline polymer can be modified through blending with chitosan. Regarding this, several studies were reported in the literature and they revealed that PVA: CS blending has the ability to be used in electrochemical devices applications including supercapacitors, solar cells, and batteries [22,23,24].

The important part to be considered in EDLC design is the polymer electrolyte separated the electrodes with conductivity between ~10^−4^ and 10^−3^ Scm^−1^ [25]. A little effort has been initiated to improve the conductivity of such polymer electrolytes, for instance salt impregnation and plasticization [26,27]. Previous studies on various polymer electrolytes revealed that glycerol plasticizer (GP) can increase the DC conductivity up to two powers [28,29,30,31,32,33]. Glycerol has multi hydroxyl (OH) moiety structure which acts as an alternative pathway for free ion from the salt to move in the polymer electrolytes [28]. Thus, GP has many OH groups to dissociate more salt and scarifying the inter and intra-hydrogen bonding that exists in the polymer matrix and consequently increasing the amorphous phases which are significant for ion transport mechanism.

In this work, PVA: CS blend is doped with 40% of ammonium thiocyanate (NH_4_SCN) and plasticized with various contents of glycerol. The purpose of this work is to use the highest conducting system of PVA: CS: NH_4_SCN: glycerol for the EDLC applications.

## 2. Materials and Methods

### 2.1. Materials

The straightforward polymeric materials are the polyvinyl alcohol (PVA) and chitosan (CS) with the average molecular weights of 310,000–375,000 g/mol, and 35,000 g/mol, respectively. Other raw materials used are: Ammonium thiocyanate (NH_4_SCN) salt was used as a dopant; acetic acid (CH_3_COOH) solution was used as a solvent; and glycerol (C_3_H_8_O_3_) was used as a plasticizer. All the chemical materials were from the commercial supplier Sigma-Aldrich (St. Louis, MO, USA).

### 2.2. Preparation of Samples

The fabrication of the PVA: CS: NH_4_SCN: glycerol based electrolyte was carried out using a solution casting technique. For this reason, 0.5 g of PVA was dissolved in 30 mL of distilled water at 80 °C for several hours. In the meantime, 0.5 g of CS was dissolved in 30 mL of 1% acetic acid solution for some hours at the ambient temperature. After cooling the PVA solution to room temperature, the separate solutions were mixed to prepare blending solution of PVA: CS polymers. Then, a constant weight percentage (40 wt.%) of ammonium thiocyanate (NH_4_SCN) was dissolved in the four individual solutions of the PVA:CS mixture. In each section of the procedure, the magnetic stirrer has been employed continually so as to produce a homogenous solution. Finally, various content of glycerol was added to the PVA: CS: 40% NH_4_SCN systems. In the prepared samples, the glycerol content was varied from 0 to 42 wt.%. The blended polymer electrolyte films were specified as PVCH0, PVCH1, PVCH2, and PVCH3 incorporated with 0, 14, 28, and 42 weight percent of glycerol, respectively. To obtain dry and free-standing films, the solutions were cast into different labeled glass Petri dishes and kept untouched to evaporate slowly at room temperature. The composition and designation of SPE samples are tabulated in Table 1.

### 2.3. Characterization of Samples

#### 2.3.1. XRD Pattern and Impedance Spectroscopy

To examine the structural changes of the prepared samples, the XRD has been employed. The Siemens D-5000 X-ray diffractometer (Bruker AXS GmbH, Berlin, Germany) was used with an operating voltage of 40 kV and a current of 40 mA. The blended SPE samples were studied through the monochromic XRD of wavelength (λ = 1.5406 Å) with a 2θ glancing angle ranging from 10° to 80° with a step size of 0.1°. The measurements of electrical impedance spectroscopy of the fabricated samples were done using an impedance analyzer of LCR meter (HIOKI 3531 Z Hi-tester, Nagano, Japan). The operating frequencies were in the range of (50 Hz ≤ *f* ≤ 1 MHz) at the room temperature. Two stainless-steel electrodes were used as a working electrode to investigate the electrolytes after the films were cut into appropriate size.

#### 2.3.2. TNM and LSV Analysis

Two of the uppermost conducting samples were characterized by the V&A instrument DP3003 digital DC power supply (V & A Instrument, Shanghai, China) to evaluate the ionic transference (*t_ion_*), and electronic transference (*t_el_*) number. The cell that consists of stainless-steel electrodes sandwiched with ion conducting film was polarized with a fixed DC potential (0.8 V). The maximum operating voltage of the uppermost conducting sample was also determined via LSV technique. The LSV was performed utilizing the Digi-IVY DY2300 potentiostat (Neware, Shenzhen, China) at a scan rate of 10 mV s^−1^ from the potential range of 0 to 2.5 V. Consequently, the cell for the LSV evaluation was prepared by means of placed the free-standing film between a pair of stainless-steel electrodes. 

#### 2.3.3. Construction of EDLC

The electrochemical double-layer capacitor EDLC has been constructed with some steps. In the first step, 6.25% of carbon black (CB), and 81.25% of activated carbon (AC) were dried and merged by planetary ball miller for less than a half-hour at 500 r/min. In the next step, 12.5% of polyvinylidene fluoride (PVdF) like a binder was dissolved in the N-methyl pyrrolidone (NMP) solution. The mixed powder of AC and CB was poured in the NMP-PVdF solution and stirred continuously until producing a black solution. Afterward, acetone was used to clean the aluminum foil and compressed on the surface of the glass. The solution mixture was cast and spread on the aluminum foil via the doctor blade method. Eventually, an oven was employed to dry the electrodes at 60 °C for some hours. For future drying, the electrodes were placed in a desiccator to eradicate any extra moisture. Also, the dried anodes were cut into a little hover shape with an area of 2.01 cm^2^. Then the most conducting sample was located among two activated carbon electrodes, and the CR2032 coin cell was used to pack the sample in a Teflon case. The CV of an EDLC was performed utilizing the Digi-IVY DY2300 potentiostat in the potential range of 0 to 0.9 V at various scan rates. The Neware battery cycler with a current density of 0.5 mA/cm^2^ was used to evaluate the charge-discharge profiles of EDLC.

## 3. Results and Discussion

### 3.1. XRD Analysis

**Figure 1:**
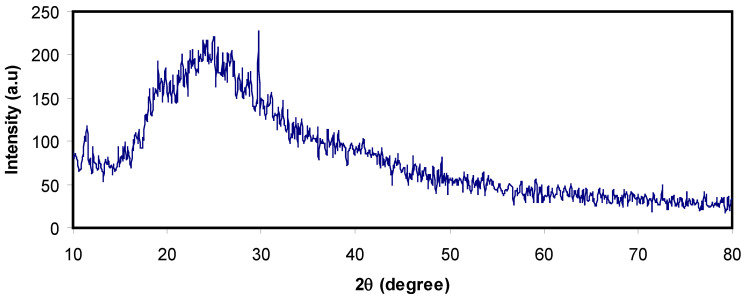
XRD spectra for the un-plasticized of the PVA: CS: 40%NH_4_SCN system at room temperature.

**Figure 2:**
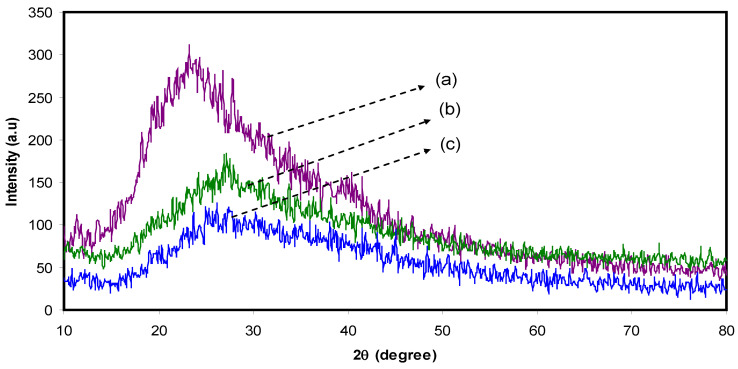
XRD patterns of plasticized PVA: CS: 40%NH_4_SCN systems incorporated with (**a**) 14 wt.% glycerol, (**b**) 28 wt.% glycerol, and (**c**) 42 wt.% glycerol, at room temperature.

**Scheme 1:**
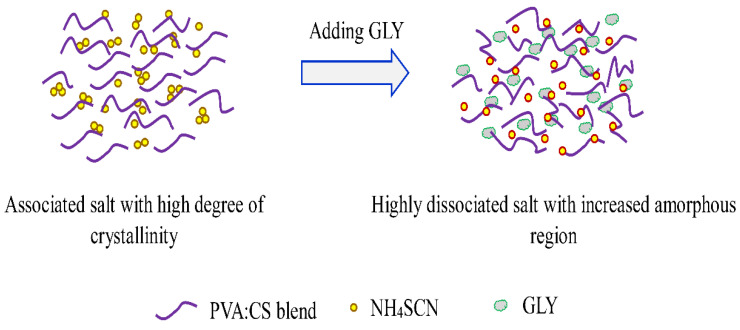
Role of plasticizer on the ion dissociation and increase of disorder.

X-ray diffraction patterns were recorded for understanding the structural changes of PVA: CS: NH_4_SCN system upon the addition of glycerol plasticizer. The diffractogram of the un-plasticized film as depicted in Figure 1 displays a crystalline narrow peak that belongs to the NH_4_SCN added salt at (2θ = 29.9°) [34,35]. It is obvious that two broad bands can be observed in the diffractogram of the un-plasticized sample at the regions 2θ of 18° and 24°. Figure 2 illustrates the XRD patterns of plasticized systems at room temperature. The glycerol content was varied from 14, to 42 weight percentages (wt.%). After the addition of glycerol, one can see that the crystalline peak disappeared, and broad hallo peaks were observed. This represents an almost complete amorphous structure because of the weakening of the intermolecular forces inside the PVA: CS polymer electrolytes [36]. Figure 2c shows that the addition of 42 wt.% of glycerol causes the decrease of the peak intensities, and results in less sharp peaks. However, the peaks experienced broadening upon the increment of the glycerol concentration. This shows the entire dissociation of NH_4_SCN salt in the amorphous area of the PVA: CS blended polymeric chain [37]. Schematically the role of glycerol plasticizer on ion dissociation and increase of amorphous phase is shown in Scheme 1. Earlier studies observed that upon addition of various plasticizers amorphous humps are enhanced and they attributed to the disruption of hydrogen bonds that built up the crystalline phase through the polymer host [29,32,33,38]. The ionic conductivity is robustly affected by the nature of crystallinity, and the amorphousness of the materials [39]. Remarkably, the ionic conductivity of the PVCH films expected to be enhanced when the amorphous nature was increased [40]. This may be ascribed to the fact that in the amorphous region, the quick segmental motions of polymeric backbone increase the charge transporter’s mobility, promoting the ionic conductivity [41].

### 3.2. EIS Study

**Figure 3:**
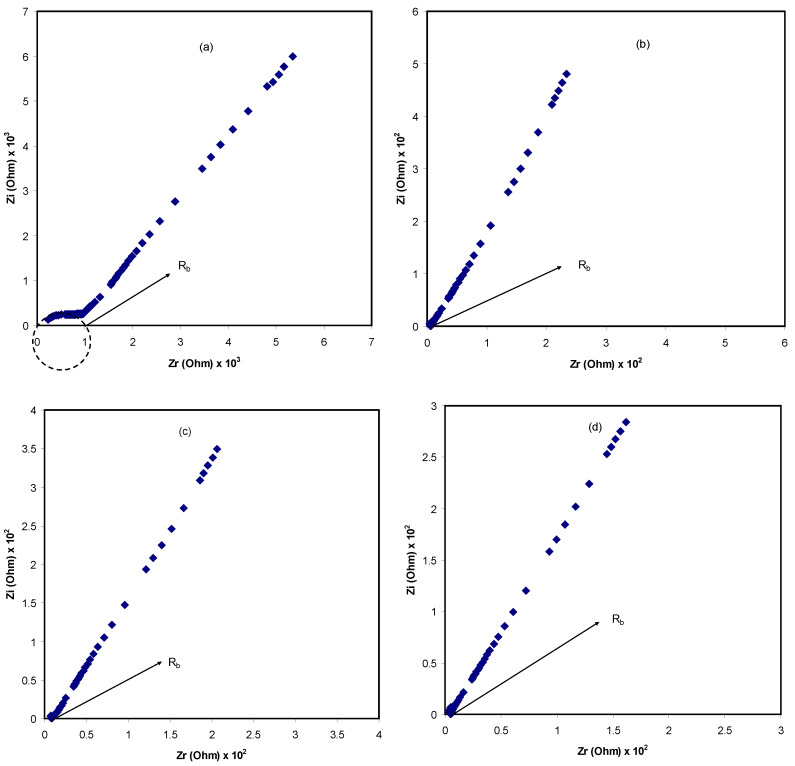
Impedance plot (*Z_i_* versus *Z_r_*) of the PVA: CS: 40%NH_4_SCN incorporated with (**a**) 0% glycerol, (**b**) 14% glycerol, (**c**) 28% glycerol, and (**d**) 42% glycerol.

In ion-conducting electrolyte materials, which are decisive from both of the technological and fundamental aspects, it is significant to grasp the mechanism of charge transport and electrical properties. In this regard, one of the influential techniques is the examination of impedance spectroscopy to illustrate the transport process and establish the relationships between structure and property [42,43]. It is well-known that the conductivity of the polymer electrolytes greatly depends on the actual density of conducting ion species and their mobilities. The impedance spectra (*Z_i_* versus *Z_r_*) of glycerol plasticized solid polymer blend electrolytes based on PVA: CS: NH_4_SCN at room temperature are depicted in Figure 3a–d. Usually, two well-defined regions are observable in the plots that include a lower-frequency inclined spike, and high-frequency semicircle. The semicircle that exists at the high frequency regions embodies the bulk effect of the electrolyte samples. While the polarization effect and the surface inhomogeneity between the electrodes and electrolyte interfaces are ascribed by the inclined spike at a lower-frequency region [44,45,46]. It can be denoted that the semicircle was observed in the un-plasticized electrolyte system of PVA: CS incorporated with 40 wt.% of NH_4_SCN while the semicircle disappeared upon adding glycerol plasticizer along with the entire experimental frequency range [47].

The bulk resistance *R_b_* values were given by the point where the semicircle intersects the horizontal axis (*Zr*-axis). Also, the ionic conductivity ($\sigma_{dc}$) was determined for the PVCH electrolyte films through the known *R_b_* value along with the sample’s dimensions using the relation below:

(1) $$\sigma_{dc}=\;\left[\frac{1}{R_{b}}\right]\;\times \;\left[\frac{t}{A}\right]$$

where *R_b_* stands for the bulk resistance, *t* is the film thickness, and *A* is equal to the known electrode area [48,49,50,51]. It was discovered that the un-plasticized system has the ionic conductivity of 10^−5^ S/cm, while it increases to 10^−3^ S/cm through adding 14 wt.% of glycerol (see Table 2). Clearly, upon an increment of the glycerol concentration in the system, the ionic conductivity was significantly improved because of the increase in the mobility of the charge carriers [52]. The glycerol has a great impact on the enhancement of ionic conductivity by promoting the salt dissociation through weakening the Coulombic force between the opposite charges [53]. Rathod et al. [54] recognized the room-temperature ionic conductivity as 3 × 10^−6^ S/cm for the PVA: CS: 20% LiClO_4_ system. Also, the PVA: CS: NH_4_I system showed the maximum conductivity of 7.6 × 10^−7^ S/cm [23]. Benitez et al. [55] investigated the polymer electrolyte system of the PVA: CS incorporated with 50% of hypophosphorous acid (H_3_PO_2_), and the obtained maximum conductivity has found to be 1.4 × 10^−2^ S/cm. Accordingly, Hamsan et al. [56] prepared the glycerol plasticized of the PS: MC: NH_4_NO_3_ based polymer electrolytes and recorded the maximum DC conductivity of ~10^−3^ S/cm. It is noteworthy that the obtained maximum conductivity in this work is comparable with the above-mentioned systems reported in the literature.

### 3.3. TNM Study

**Figure 4:**
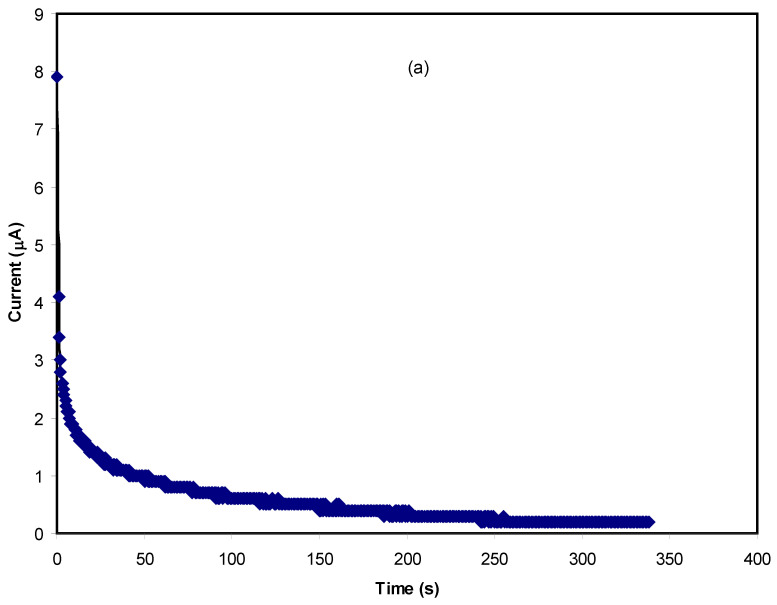

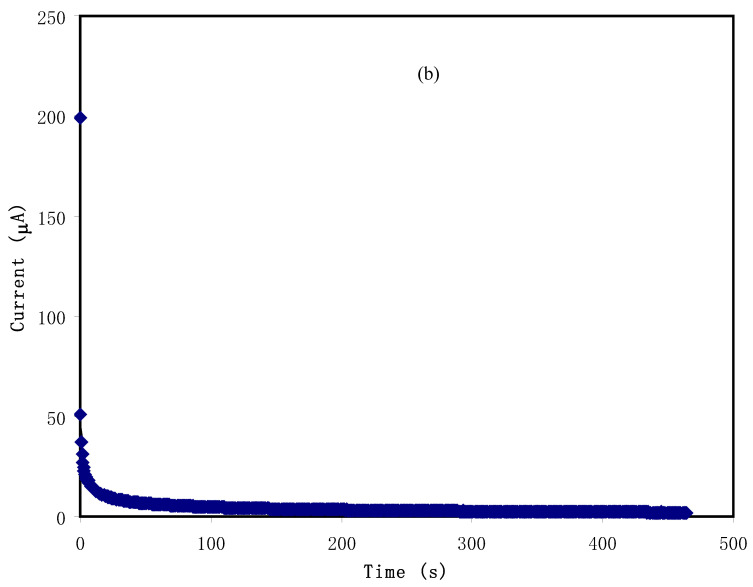
Current against time for the (**a**) PVCH2, and (**b**) PVCH3 electrolyte films.

The conductivity in the polymer electrolyte is given mainly by the conduction of ions and insignificantly by electrons. Electrolytes are considered to be the heart of electrochemical devices, and their ionic conductivity examination is significant from the perspective of device applications such as supercapacitor and battery [57]. The TNM has been employed to figure out the main charge carrier at the working voltage of 0.2 V. Figure 4a,b displays the polarization curve of current against time for the two of the most conducting samples (i.e., PVCH2 and PVCH3) at ambient temperature, respectively. Figure 4a belongs to 28 wt.% of added glycerol to the PVA: CS: 40%NH_4_SCN system, and shows a current of (7.9 µA) at the TNM initiation. Therefore, an extreme drop can be seen until the time of 30 s, even though the current is continued to decrease gradually up until the time of 250 s. On the other hand, the polarization curve of the uppermost conducting sample (i.e., PVCH3) reveals a very high initial current value of 200 µA. A rapid initial current can be observed with time up to 20 s, subsequently, the current slowly moved to stabilize at about 0.1 µA. The drop of the initial total current versus time is mainly due to the lessening of the ionic charge carrier species in the electrolyte systems [58,59]. Based on the TNM plots, the ions and electrons transference number were evaluated using the following equations:

(2) $$t_{ion}=\frac{I_{i}-I_{SS}}{I_{i}}$$

(3) $$t_{ion}=1-t_{el}$$

where *t_ion_* is the ion transport, *t_el_* stands for the electron transport, *I_i_* stands for the initial current which embraces’ electrons and ions, and *I_ss_* is the steady-state current holds electron only. The *t_ion_* result outcomes value was found to be 0.97 and 0.989 for the samples of PVCH2, and PVCH3, respectively. The prepared PVCH3 system is purely ionic because the *t_ion_* value is ~1. This indicates that the charge immigration in the SPE is mainly as a result of ions [60]. Our previous work for the glycerolized CS: NH_4_F system possesses 0.976 of *t_ion_* value, which is in good agreement with the current study [38].

### 3.4. LSV Study

**Figure 5:**
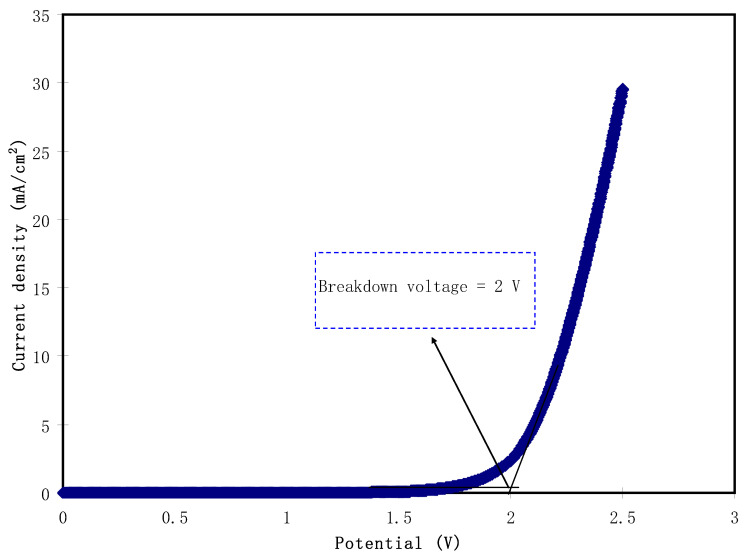
Linear sweep voltammogram for the PVCH3 electrolyte sample at room temperature.

The analysis of electrochemical stability is central for the real applications of the electrochemical devices. LSV has been engaged to specify the maximum operating voltage of the uppermost conducting sample at the scan rate of 10 mV s^−1^ mV/s [61]. Figure 5 represents a potential window of the PVCH3 blend polymer electrolyte film at ambient temperature. An applied voltage has been measured in the range of 0 to 2.5 V using two stainless-steel working electrodes [62]. It was found that there is no residual electronic current up to 1.75 V, and the breakdown voltage of the PVA: CS: 40%NH_4_SCN: 42%glycerol system was found to be ~2 V. Beyond 2 V the drastic rise in the current density is observable, which signifies the breakdown of the film at the inert electrode surface. Consequently, the prepared PVCH3-based SPE has adequate anode stability that can be used as electrode separators in the application of electrochemical devices [63,64]. The result is analogous with other glycerol plasticizer and ammonium salt-based blend solid polymer electrolytes. The system including methylcellulose/potato starch (MC:PS) integrated with ammonium nitrate (NH_4_NO_3_) and plasticized with glycerol reported by Hamsan et al. [65] has the breakdown voltage of 1.88 V. Yusof et al. [66] documented the decomposition voltage of 1.9 V for glycerol plasticized of the starch-chitosan-NH_4_I system. Obviously, their results were found to be lower than the outcome obtained in this work. 

### 3.5. CV Study

**Figure 6:**
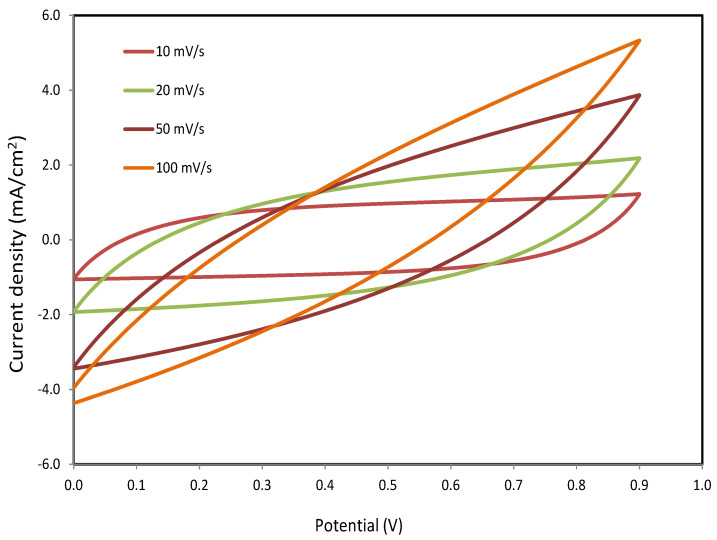
Cyclic voltammogram responses of the EDLC fabrication for the PVCH3 at different scan rates in the potential rage of 0 to 0.9 V.

The electrochemical routines of the prepared PVCH3-based solid polymer electrolyte system has been investigated using CV technique. The CV investigation of the fabricated device is vital to be measured in order to use the device for EDLC application [67]. The CV curve of the maximum ion-conducting sample in a potential window ranging from 0 to 0.9 V at various scan rates of 10–100 mV/s is depicted in Figure 6 at room temperature. Overall, the leaf shape can be seen for all the CV plots, and the specific capacitance of the fabricated EDLC has been determined via the following equation:

(4) $$C_{CV}=\;\int_{V_{i}}^{V_{f}}\frac{I\left(V\right)dV}{2ma\;\left(V_{f}-V_{i}\right)}$$

where $V_{i\;}$ and $V_{f}$ are the initial and final voltages, respectively. m stands for the mass of active material, *υ* stands for the scan rates, and ∫(V)dV is the real area of the CV curve. The rectangular-like shapes of cyclic voltammogram were obtained once the capacitance is potential independent, whereas if the capacitance relies on voltage the state of cyclic voltammogram displays an alternate profile [68,69,70]. The highest specific capacitance *C_s_* for the sample that is incorporated with 42 wt.% of glycerol was calculated based on Equation (4), and found to be 25.05 F/g, at the scan rate of 10 mV/s [71]. It is also noticeable that the specific capacitance *C_s_* values varied with changing the scan rates, as shown in Table 3. Furthermore, the values of specific capacitance decrease with increasing scan rates because of the unreachable portion for ions dispersion at the interfaces between the electrodes and electrolyte sample. It can be said that the PVCH3 electrolyte film admirably has electrochemical stability because of the almost nearly rectangular mirror images of the shape of the voltammograms, especially at a high sweep rate. Meanwhile, the CV curve shows an ideal capacitor since the plot possesses no visible peaks in the polarization curve, which means no redox reaction occurred with the voltage of 0.9 V [72,73,74]. The specific capacitance *C_s_* value of fabricated EDLC achieved in this work was found to be higher than some previously reported works. Shukur et al. [75] fabricated EDLC with activated carbon using CS: NH_4_Br: Glycerol, which obtained 7.5 F/g from CV analysis at scan rate of 10 mV/s. Also, the C_s_ value of our previous study for the CS: PEO: NH_4_SCN system was 3.8 F/g at 50 mV/s [4]. 

### 3.6. Charge-Discharge Study

**Figure 7:**
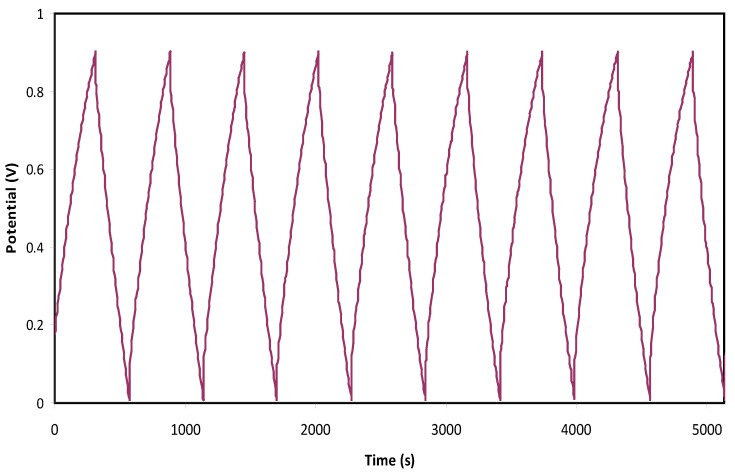
Charge-discharge profile of the fabricated EDLC at selected cycles.

Figure 7 demonstrates the typical galvanostatic charge-discharge (GCD) curve of constructed EDLC over 150 cycles at a constant current density of 0.5 mA/cm^2^ in the potential ranging from 0 to 0.9 V at room temperature. One can see that these nine cycles indicate the outstanding capacitive performance of EDLC because of their shape of almost perfect symmetrical patterns [76]. However, a slight deviation can be observed in the voltage drop of discharge characteristics from their triangle-like shapes attributed to the roughness of activated carbon, internal resistance, and bulk electrolyte. The discharge slop linearity represents the relatively pure electrostatic interactions between the ions and charged pore surface [77]. Furthermore, the existence of the non-Faradaic charge storage process can be recognized through symmetric triangles of the GCD curve [78].

**Figure 8:**
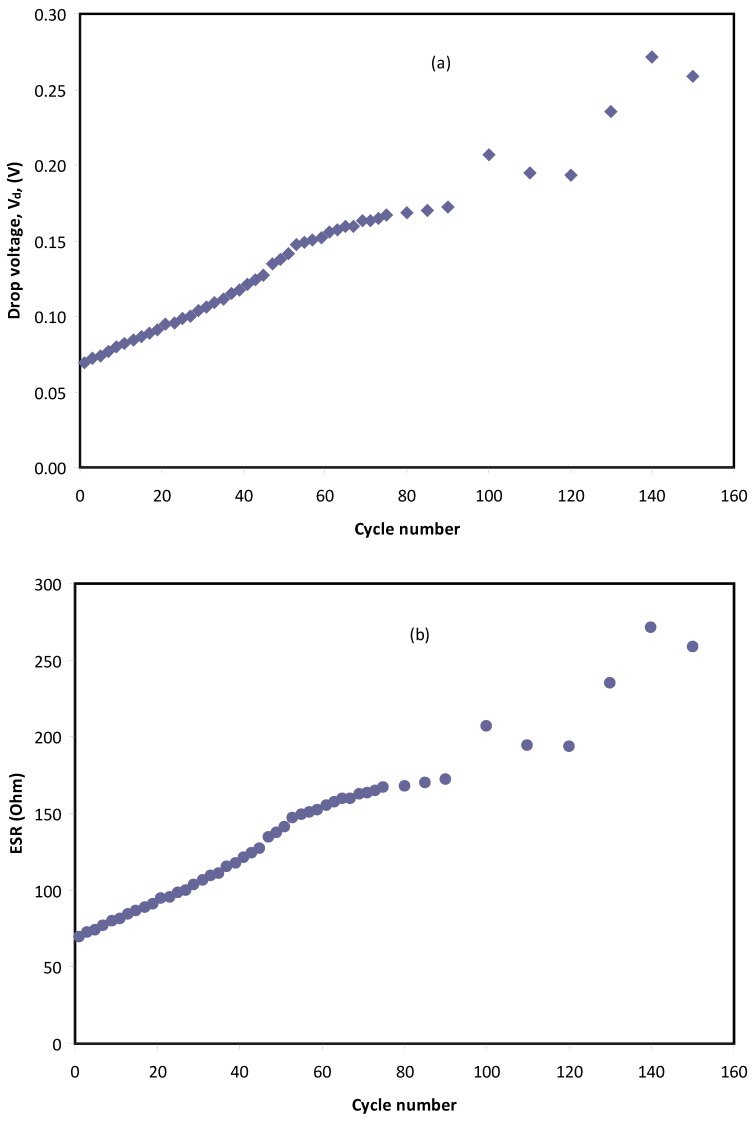
The EDLC parameters of (**a**) drop voltage (*V_d_*) of discharge process initiation, (**b**) equivalent series resistance (ESR) pattern up to 150th cycles.

The drop voltage (*V_d_*) of the constructed EDLC and equivalent series resistance (*R_es_*) versus cycle number are presented in Figure 8a,b, respectively. The value of drop voltage V_d_ acquired for the first cycle was found to be about 0.07 V, while it gradually increased over the cycles beyond 120th. Afterthought, the drop potential is raised and attained the maximum value (i.e., 0.28 V) at the 140th cycle. This phenomenon takes place because of the increment in the internal resistance which is known as the equivalent series resistance *R_es_* that can be determined from the following equation:

(5) $$R_{es}=\;\frac{V_{d}}{i}$$

where *V_d_* stands for the drop voltage before the discharge process initiates, and *i* is the applied current [79]. From Figure 8b, the ESR value at the first cycle was found to be 65 Ω. It can also be observed that the values of (*R_es_*) increase with increasing the cycle number and reached the maximum value of approximately 275 Ω at the cycle number of 140 because of the voltage drop, *V_d_* is the highest value. In the fabricated EDLC, the presence of internal resistance is mainly attributed to the electrolyte used for the current collector, and the gap between electrolyte with current collector [80,81]. In this investigation, the result of (*R_es_*) for the manufactured EDLC is tolerable and comparable to some other previously reported activated carbon-based EDLC works [76,79,82].

**Figure 9:**
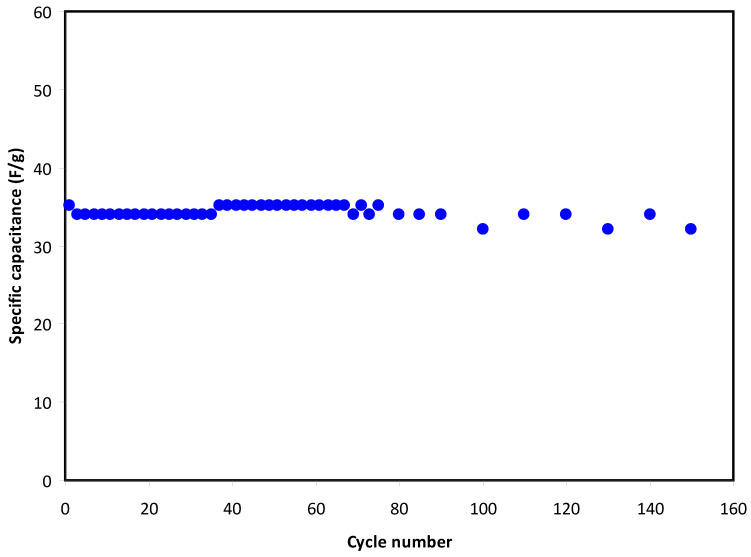
The specific capacitance, *C_s_* of the constructed EDLC versus cycle number.

The variation of the specific capacitance, *C_s_* of the fabricated EDLC for the 150th cycles is presented in Figure 9. The *C_s_* value of the constructed EDLC was determined via the equation below:

(6) $$C_{s}=\frac{i}{gm}$$

where *i* is the applied current, *g* stands for the gradient of discharge fragment, and *m* is the weight of active material. It is worth noting that the first cycle discharge *C_s_* value was found to be 35.5 F/g, and followed by a slight decrease at the 150th cycle to 34 F/g, which may be owing to the decomposition of the prepared polymer electrolyte [83]. An interesting observation is that the specific capacitance *C_s_* values are almost constant over 150 charge-discharge cycles, which verify its perfect cycling stability [84,85]. Moreover, the low lattice energy of the salt is noteworthy as enhancing the specific capacitance value, which possesses high dissociation [86]. The obtained *C_s_* value in our previous study for the CS: Dextrane: NH_4_F system was 12.4 F/g, which is lower than the present *C_s_* value [87]. The *C_s_* pattern in our previous study for CS: Dextran: NH_4_I is completely different from the results of the *C_s_* pattern present work. This is may be due to the lattice energy of NH_4_SCN salt is lower compared to NH_4_F. Thus, the strong interaction between NH_4_^+^ with F^−^ is predictable than NH_4_^+^ with SCN^−^. Compared to Figure 8, the trend of *C_s_* with cycle number is different. The ESR is calculated from drop voltage. With increasing cycle number, the drop voltage also increased. Thus ESR, which is proportional to drop voltage, also rises with increasing cycle number. On the other hand in the equation of specific capacitance the drop voltage is absent. Thus its trend is different. Usually the slop of linear part of discharge curve excluding the drop voltage was used to calculate the slop (g) in Equation (6).

**Figure 10:**
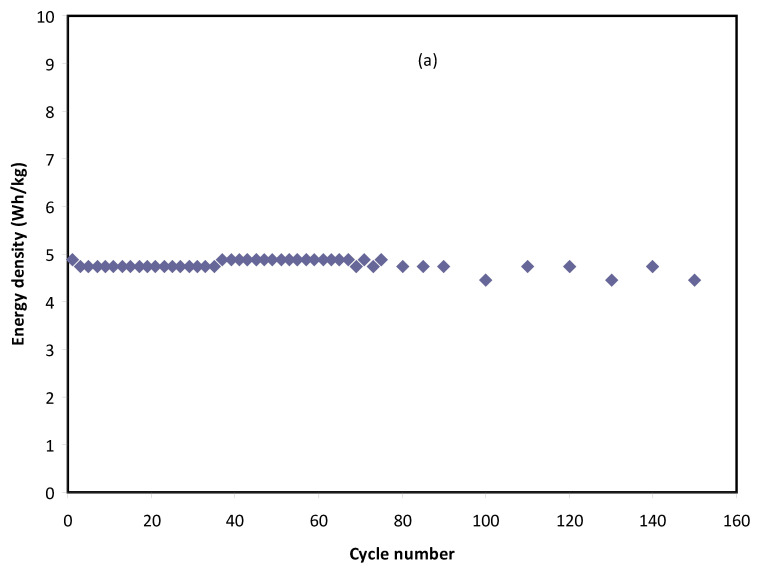

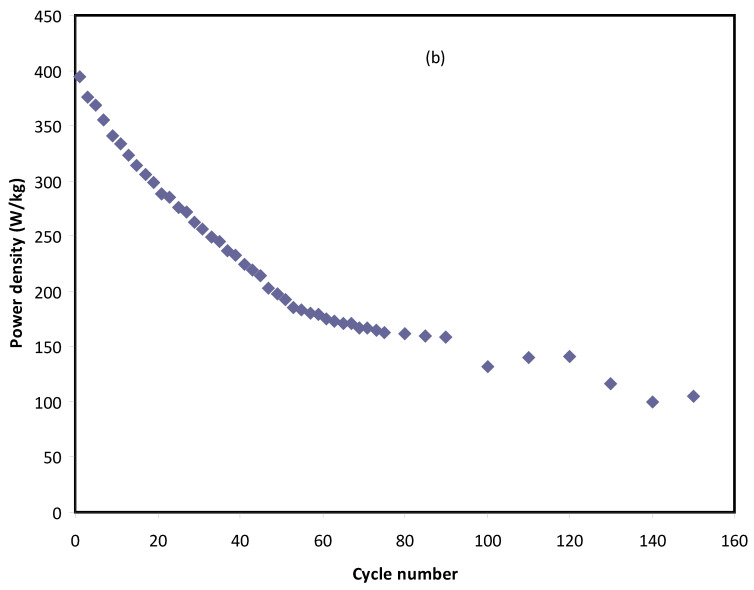
The EDLC parameters of (**a**) energy density (*E_d_*), and (**b**) power density (*P_d_*) at selected cycles.

There are two more significant parameters that have to be determined for the EDLC, which are: energy (*E_d_*, Wh/kg) and power (*P_d_*, W/kg) densities to confirm the EDLC practical device applications. Figure 10a and b demonstrate the values of energy density (*E_d_*) and power density (*P_d_*) at selected cycles for the PVA: CS: NH_4_SCN:glycerol-based EDLC, respectively [88]. Both the EDLC parameters value of energy density (*E_d_*) and power density (*P_d_*) were calculated using the following equations [79]:

(7) $$E_{d}=\frac{C_{S}V^{2}}{2}$$

(8) $$P_{d}=\frac{V^{2}}{4m\left(R_{es}\right)}$$

where *C_s_* stands for the specific capacitance, *V* is the applied voltage, *m* and *R_es_* are the weight of active material and equivalent series resistance, respectively. It can be seen that the *E_d_* value at the first cycle is 4.9 Wh/kg, and beyond it just about unchanged toward the 150th cycle with an average of ~4.8 Wh/kg (see Figure 10a). This display implies the almost same energy barrier for the transportation of ions in the prepared electrolyte film, from the 1st to 150th cycle [89]. Pandey et al. [88] have stated the energy density, *E_d_* of (0.3 Wh/kg) in their electrolyte system, which is worse than the current work. Additionally, the outcome of energy density, *E_d_* achieved in this work is near to the reported (5.5 Wh/kg) one for the based gel polymer electrolyte [90].

From Figure 10b, it is predictable that the power density, *P_d_* decreased with the increment of the cycle number because of the increase of drop voltage and *ESR* (see Figure 8a). The power density, *P_d_* value for the first cycle was found to be 399 W/kg. It also dropped to about 180 W/kg at the 50th cycle with continuously released to 100 W/kg, once the EDLC finalized the 150 cycles. The drop in *P_d_* value is due to ion accumulation from the recombination of charge species during a quick charge-discharge procedure, which blocks the transport of ions [91]. Liew et al. [92] documented that the *P_d_* value of 18.37 W/kg obtained in the first cycle using the PVA-CH_3_COONH_4_-BmImTf system, is obviously lower than this study.

## 4. Conclusions

In conclusion, the plasticized polymer blend electrolytes have been prepared via solution-casting method. Polyvinyl alcohol (PVA) and chitosan (CS) were used as the basic polymeric materials. Ion-conducting carriers from ammonium thiocyanate (NH_4_SCN) and glycerol plasticizer were used to increase the number of free ions and enhance the amorphous phase. The XRD results confirmed the role of plasticizer to enrich the amorphous content. Appearance of broad humps and disappearance of the crystalline sharp peaks in plasticized systems established that plasticization is a novel approach to improve the performance of polymer electrolytes for device application. Through the EIS technique, the sample incorporated with 42 wt.% of plasticizer possesses the maximum conductivity of 3.5 × 10^−3^ S/cm with the electrochemical stability window up to 2 V. The results from TNM analysis for the two of the highest conducting samples portrayed that the ions in the PVCH systems were the dominant charge carriers. The uppermost conducting sample (i.e., PVCH3) verified that it can be employed in the preparation of the EDLC. The (CV) plot displays an ideal capacitor since the EDLC did not experience either reduction or oxidation reaction. The constructed EDLC offers an initial specific capacitance (*C_s_*) of 35.5 F/g, equivalent series resistance (*R_es_*) of 65 Ω, energy density (*E_d_*) of 4.9 Wh/kg, and power density (*P_d_*) of 399 W/kg. In the present study, the result outcomes imply that the PVCH electrolyte systems have the potential to be used in EDLC devices.

## Figures and Tables

**Table 1:** The composition and designation of the PVA: CS: NH_4_SCN: glycrol systems.

Sample Designation	PVA:CS (g)	NH_4_SCN (g)	Glycerol (wt.%)	Glycerol (g)
PVCH0	0.5:0.5	0.666	0	0
PVCH1	0.5:0.5	0.666	14	0.271
PVCH2	0.5:0.5	0.666	28	0.647
PVCH3	0.5:0.5	0.666	42	1.206

**Table 2:** The ionic conductivity $\sigma_{dc}$ of un-plasticized and plasticized of PVA: CS: NH_4_SCN systems at ambient temperature.

Sample Code	Ionic Conductivity *σ_dc_* (S cm^−1^)	Bulk Resistance (R_b_) (Ohm)
PVCH0	1.4 × 10^−5^	995
PVCH1	1.4 × 10^−3^	9.8
PVCH2	2 × 10^−3^	7.1
PVCH3	3.5 × 10^−3^	3.9

**Table 3:** The specific capacitance (F/g) value of PVCH3 sample at the scan rates of 10, 20, 50, and 100 mV s^−1.^

Scan Rate (mV/s)	Specific Capacitance (F/g)
10	25.052
20	17.962
50	7.994
100	3.763

## References

[1] Staiti P., Minutoli M., Lufrano F. (2002). All solid electric double layer capacitors based on Nafion ionomer. Electrochim. Acta.

[2] Wei L., Yushin G. (2012). Nanostructured activated carbons from natural precursors for electrical double layer capacitors. Nano Energy.

[3] Sato T., Marukane S., Morinaga T., Kamijo T., Arafune H., Tsujii Y. (2015). High voltage electric double layer capacitor using a novel solid-state polymer electrolyte. J. Power Sources.

[4] Aziz S., Abdulwahid R., Hamsan M.H., Brza M.A., Abdullah R.M., Kadir M., Muzakir S.K. (2019). Structural, Impedance, and EDLC Characteristics of Proton Conducting Chitosan-Based Polymer Blend Electrolytes with High Electrochemical Stability. Molecules.

[5] Wei L., Sevilla M., Fuertes A.B., Mokaya R., Yushin G., Valdés-Solís T. (2011). Polypyrrole-Derived Activated Carbons for High-Performance Electrical Double-Layer Capacitors with Ionic Liquid Electrolyte. Adv. Funct. Mater..

[6] Zhao X.Y., Wu Y., Cao J.P., Zhuang Q.Q., Wan X., He S., Wei X.Y. (2018). Preparation and characterization of activated carbons from oxygen-rich lignite for electric double-layer capacitor. Int. J. Electrochem. Sci..

[7] Aziz S., Hamsan M.H., Kadir M.F.Z., Karim W., Abdullah R.M. (2019). Development of Polymer Blend Electrolyte Membranes Based on Chitosan: Dextran with High Ion Transport Properties for EDLC Application. Int. J. Mol. Sci..

[8] Hashim M.A., Sa’Adu L. (2014). A Flexible Solid State EDLC from a Commercially Prepared Multiwalled Carbon Nanotubes and Hybrid Polymer Electrolytes. J. Mater. Sci. Res..

[9] Das S., Ghosh A. (2017). Solid Polymer Electrolyte Based on PVDF-HFP and Ionic Liquid Embedded with TiO2Nanoparticle for Electric Double Layer Capacitor (EDLC) Application. J. Electrochem. Soc..

[10] Verma M.L., Minakshi M., Singh N.K. (2014). Structural and Electrochemical Properties of Nanocomposite Polymer Electrolyte for Electrochemical Devices. Ind. Eng. Chem. Res..

[11] Verma M.L., Minakshi M., Singh N.K. (2014). Synthesis and Characterization of Solid Polymer Electrolyte based on Activated Carbon for Solid State Capacitor. Electrochim. Acta.

[12] Rodríguez-Moreno J., Navarrete-Astorga E., Dalchiele E.A., Sánchez L., Ramos-Barrado J., Martín F. (2013). Polyvinylpyrrolidone-LiClO4 solid polymer electrolyte and its application in transparent thin film supercapacitors. J. Power Sources.

[13] Aziz S.B. (2016). Structural, Morphological and Electrochemical Impedance Study of CS: LiTf based Solid Polymer Electrolyte: Reformulated Arrhenius Equation for Ion Transport Study. Int. J. Electrochem. Sci..

[14] Shukur M.F., Yusof Y.M., Zawawi S.M.M., Illias H.A., Kadir M. (2013). Conductivity and transport studies of plasticized chitosan-based proton conducting biopolymer electrolytes. Phys. Scr..

[15] Aziz S., Abdullah O.G., Hussein S.A. (2018). Role of Silver Salts Lattice Energy on Conductivity Drops in Chitosan Based Solid Electrolyte: Structural, Morphological and Electrical Characteristics. J. Electron. Mater..

[16] Ramkumar R., Minakshi M. (2015). Fabrication of ultrathin CoMoO 4 nanosheets modified with chitosan and their improved performance in energy storage device. Dalton Trans..

[17] Ramkumar R., Sundaram M.M. (2016). A biopolymer gel-decorated cobalt molybdate nanowafer: Effective graft polymer cross-linked with an organic acid for better energy storage. New J. Chem..

[18] Yang C.-C., Lin S.-J., Wu G.-M. (2005). Study of ionic transport properties of alkaline poly(vinyl) alcohol-based polymer electrolytes. Mater. Chem. Phys..

[19] Aziz S., Abdullah R.M., Rasheed M.A., Ahmed H.M. (2017). Role of Ion Dissociation on DC Conductivity and Silver Nanoparticle Formation in PVA:AgNt Based Polymer Electrolytes: Deep Insights to Ion Transport Mechanism. Polymers.

[20] Osman Z., Ibrahim Z., Arof A. (2001). Conductivity enhancement due to ion dissociation in plasticized chitosan based polymer electrolytes. Carbohydr. Polym..

[21] Aziz S. (2016). Role of Dielectric Constant on Ion Transport: Reformulated Arrhenius Equation. Adv. Mater. Sci. Eng..

[22] Kadir M., Aspanut Z., Majid S., Arof A.K. (2011). FTIR studies of plasticized poly(vinyl alcohol)-chitosan blend doped with NH4NO3 polymer electrolyte membrane. Spectrochim. Acta Part A Mol. Biomol. Spectrosc..

[23] Marf A.S., Abdullah R.M., Aziz S.B. (2020). Structural, Morphological, Electrical and Electrochemical Properties of PVA: CS-Based Proton-Conducting Polymer Blend Electrolytes. Membranes.

[24] Kadir M., Majid S., Arof A.K. (2010). Plasticized chitosan-PVA blend polymer electrolyte based proton battery. Electrochim. Acta.

[25] Poonam K., Sharma A., Arora S.K. (2019). Review of supercapacitors: Materials and devices. J. Energy Storage.

[26] Ibrahim S., Yasin S.M.M., Nee N.M., Ahmad R., Johan M.R. (2012). Conductivity and dielectric behaviour of PEO-based solid nanocomposite polymer electrolytes. Solid State Commun..

[27] Aziz S., Woo T.J., Kadir M.F., Ahmed H.M., Ahmed H.M. (2018). A conceptual review on polymer electrolytes and ion transport models. J. Sci. Adv. Mater. Devices.

[28] Hamsan H., Aziz S.B., Kadir M., Brza M., Karim W.O. (2020). The study of EDLC device fabricated from plasticized magnesium ion conducting chitosan based polymer electrolyte. Polym. Test..

[29] Asnawi A.S.F.M., Aziz S., Nofal M., Hamsan M.H., Brza M.A., Yusof Y.M., Abdulwahid R., Muzakir S.K., Kadir M. (2020). Glycerolized Li^+^ Ion Conducting Chitosan-Based Polymer Electrolyte for Energy Storage EDLC Device Applications with Relatively High Energy Density. Polymers.

[30] Hamsan M., Aziz S.B., Azha M., Azli A., Shukur M., Yusof Y., Muzakir S., Manan N.S., Kadir M. (2020). Solid-state double layer capacitors and protonic cell fabricated with dextran from Leuconostoc mesenteroides based green polymer electrolyte. Mater. Chem. Phys..

[31] Sekhar P.C. (2012). Effect of plasticizer on conductivity and cell parameters of (PMMA+NaClO4) polymer electrolyte system. IOSR J. Appl. Phys..

[32] Hadi J.M., Aziz S.B., Nofal M., Hussein S.A., Hafiz M.H., Brza M.A., Abdulwahid R., Kadir M., Woo H.J., Hamsan M.H. (2020). Electrical, Dielectric Property and Electrochemical Performances of Plasticized Silver Ion-Conducting Chitosan-Based Polymer Nanocomposites. Membranes.

[33] Hamsan M., Aziz S.B., Nofal M., Brza M., Abdulwahid R., Hadi J.M., Karim W.O., Kadir M. (2020). Characteristics of EDLC device fabricated from plasticized chitosan: MgCl2 based polymer electrolyte. J. Mater. Res. Technol..

[34] Shukur M.F., Kadir M. (2015). Hydrogen ion conducting starch-chitosan blend based electrolyte for application in electrochemical devices. Electrochim. Acta.

[35] Göktepe F., Çelik S.Ü., Bozkurt A. (2008). Preparation and the proton conductivity of chitosan/poly(vinyl phosphonic acid) complex polymer electrolytes. J. Non-Cryst. Solids.

[36] Aziz S., Abidin Z., Arof A.K. (2010). Effect of silver nanoparticles on the DC conductivity in chitosansilver triflate polymer electrolyte. Phys. B Condens. Matter.

[37] Aziz S., Hassan A.Q., Mohammed S.J., Karim W., Kadir M., Tajuddin H.A., Chan N.N.M.Y. (2019). Structural and Optical Characteristics of PVA:C-Dot Composites: Tuning the Absorption of Ultra Violet (UV) Region. Nanomaterials.

[38] Asnawi A.S.F.M., Aziz S., Nofal M., Yusof Y.M., Brevik I., Hamsan M.H., Brza M.A., Abdulwahid R., Kadir M. (2020). Metal Complex as a Novel Approach to Enhance the Amorphous Phase and Improve the EDLC Performance of Plasticized Proton Conducting Chitosan-Based Polymer Electrolyte. Membranes.

[39] Yulianti E., Karo A.K., Susita L., Sudaryanto (2012). Synthesis of Electrolyte Polymer Based on Natural Polymer Chitosan by Ion Implantation Technique. Procedia Chem..

[40] Mobarak N.N., Ahmad A., Abdullah M., Ramli N., Rahman M.Y.A. (2013). Conductivity enhancement via chemical modification of chitosan based green polymer electrolyte. Electrochim. Acta.

[41] Rosli N., Chan C., Subban R., Winie T. (2012). Studies on the Structural and Electrical Properties of Hexanoyl Chitosan/Polystyrene-based Polymer Electrolytes. Phys. Procedia.

[42] Machappa T., Prasad M.A. (2009). AC conductivity and dielectric behavior of polyaniline/sodium metavenadate (PANI/NaVO3) composites. Phys. B Condens. Matter.

[43] Aziz S.B., Karim W.O., Ghareeb H.O. (2020). The deficiency of chitosan:AgNO3 polymer electrolyte incorporated with titanium dioxide filler for device fabrication and membrane separation technology. J. Mater. Res. Technol..

[44] Siddaiah T., Ojha P., Gopal N., Ramu C., Nagabhushana H. (2018). Thermal, structural, optical and electrical properties of PVA/MAA:EA polymer blend filled with different concentrations of Lithium Perchlorate. J. Sci. Adv. Mater. Devices.

[45] Aziz S.B., Brza M., Saed S.R., Hamsan M., Kadir M. (2020). Ion association as a main shortcoming in polymer blend electrolytes based on CS:PS incorporated with various amounts of ammonium tetrafluoroborate. J. Mater. Res. Technol..

[46] Muchakayala R., Song S., Gao S., Wang X., Fan Y. (2017). Structure and ion transport in an ethylene carbonate-modified biodegradable gel polymer electrolyte. Polym. Test..

[47] Yusof Y.M., Illias H.A., Kadir M. (2014). Incorporation of NH4Br in PVA-chitosan blend-based polymer electrolyte and its effect on the conductivity and other electrical properties. Ionics.

[48] Polu A.R., Kumar R. (2011). AC impedance and dielectric spectroscopic studies of Mg2+ ion conducting PVA-PEG blended polymer electrolytes. Bull. Mater. Sci..

[49] Mustafa M.S., Ghareeb H.O., Aziz S., Brza M.A., Al-Zangana S., Hadi J.M., Kadir M. (2020). Electrochemical Characteristics of Glycerolized PEO-Based Polymer Electrolytes. Membranes.

[50] Hadi J.M. (2020). Electrochemical Impedance study of Proton Conducting Polymer Electrolytes based on PVC Doped with Thiocyanate and Plasticized with Glycerol. Int. J. Electrochem. Sci..

[51] Aziz S.B., Hamsan M.H.H., Nofal M., San S., Abdulwahid R., Saeed S.R.R., Brza M.A., Kadir M., Mohammed S.J., Al-Zangana S. (2020). From Cellulose, Shrimp and Crab Shells to Energy Storage EDLC Cells: The Study of Structural and Electrochemical Properties of Proton Conducting Chitosan-Based Biopolymer Blend Electrolytes. Polymers.

[52] Al-Muntaser A., Abdelghany A., Abdelrazek E., Elshahawy A. (2020). Enhancement of optical and electrical properties of PVC/PMMA blend films doped with Li4Ti5O12 nanoparticles. J. Mater. Res. Technol..

[53] Shukur M.F., Ithnin R., Kadir M. (2016). Ionic conductivity and dielectric properties of potato starch-magnesium acetate biopolymer electrolytes: The effect of glycerol and 1-butyl-3-methylimidazolium chloride. Ionics.

[54] Rathod S.G., Bhajantri R.F., Ravindrachary V., Pujari P.K., Sheela T. (2014). Ionic conductivity and dielectric studies of LiClO4 doped poly(vinylalcohol)(PVA)/chitosan(CS) composites. J. Adv. Dielectr..

[55] Benítez M., Astaiza J.D., Vargas R.A. (2018). Effect of H3PO2 on the mechanical, thermal, and electrical properties of polymers based on poly (vinyl alcohol) (PVA) and chitosan (CS). Ionics.

[56] Hamsan M.H., Shukur M.F., Kadir M. (2017). NH4NO3 as charge carrier contributor in glycerolized potato starch-methyl cellulose blend-based polymer electrolyte and the application in electrochemical double-layer capacitor. Ionics.

[57] Aziz S.B., Brza M.A., Hamsan H.M., Kadir M.F.Z., Abdulwahid R.T. (2020). Electrochemical characteristics of solid state double-layer capacitor constructed from proton conducting chitosan-based polymer blend electrolytes. Polym. Bull..

[58] Samsudin A., Lai H., Isa M.I.N. (2014). Biopolymer Materials Based Carboxymethyl Cellulose as a Proton Conducting Biopolymer Electrolyte for Application in Rechargeable Proton Battery. Electrochim. Acta.

[59] Noor N., Isa M.I.N. (2019). Investigation on transport and thermal studies of solid polymer electrolyte based on carboxymethyl cellulose doped ammonium thiocyanate for potential application in electrochemical devices. Int. J. Hydrogen Energy.

[60] Hadi J.M., Aziz S.B., Mustafa M.S., Hamsan M.H., Abdulwahid R.T., Kadir M.F., Ghareeb H.O. (2020). Role of nano-capacitor on dielectric constant enhancement in PEO:NH4SCN:xCeO2 polymer nano-composites: Electrical and electrochemical properties. J. Mater. Res. Technol..

[61] Cheng H., Zhu C., Huang B., Lu M., Yang Y. (2007). Synthesis and electrochemical characterization of PEO-based polymer electrolytes with room temperature ionic liquids. Electrochim. Acta.

[62] Francis K.A., Liew C.-W., Ramesh S., Ramesh K. (2015). Ionic liquid enhanced magnesium-based polymer electrolytes for electrical double-layer capacitors. Ionics.

[63] Dhatarwal P., Choudhary S., Sengwa R.J. (2018). Electrochemical performance of Li+-ion conducting solid polymer electrolytes based on PEO-PMMA blend matrix incorporated with various inorganic nanoparticles for the lithium ion batteries. Compos. Commun..

[64] Aziz S.B., Brza M., Mishra K., Hamsan M., Karim W.O., Abdullah R.M., Kadir M., Abdulwahid R.T. (2020). Fabrication of high performance energy storage EDLC device from proton conducting methylcellulose: Dextran polymer blend electrolytes. J. Mater. Res. Technol..

[65] Hamsan M.H., Aziz S.B., Shukur M.F., Kadir M. (2019). Protonic cell performance employing electrolytes based on plasticized methylcellulose-potato starch-NH4NO3. Ionics.

[66] Yusof Y.M., Majid N.A., Kasmani R.M., Illias H.A., Kadir M.F.Z. (2014). The Effect of Plasticization on Conductivity and Other Properties of Starch/Chitosan Blend Biopolymer Electrolyte Incorporated with Ammonium Iodide. Mol. Cryst. Liq. Cryst..

[67] Mazuki N., Majeed A.P.P.A., Samsudin A.S. (2020). Study on electrochemical properties of CMC-PVA doped NH4Br based solid polymer electrolytes system as application for EDLC. J. Polym. Res..

[68] Aziz S.B., Hamsan M.H., Abdullah R.M., Abdulwahid R.T., Brza M.A., Marif A.S., Kadir M.F.Z. (2020). Protonic EDLC cell based on chitosan (CS): Methylcellulose (MC) solid polymer blend electrolytes. Ionics.

[69] Nadiah N., Omar F.S., Numan A., Mahipal Y.K., Ramesh S., Ramesh K. (2017). Influence of acrylic acid on ethylene carbonate/dimethyl carbonate based liquid electrolyte and its supercapacitor application. Int. J. Hydrogen Energy.

[70] Bandaranayake C., Weerasinghe W., Vidanapathirana K., Perera K.S. (2016). A Cyclic Voltammetry study of a gel polymer electrolyte based redox-capacitor. Sri Lankan J. Phys..

[71] Chong M.Y., Numan A., Liew C.-W., Ng H., Ramesh K., Ramesh S. (2018). Enhancing the performance of green solid-state electric double-layer capacitor incorporated with fumed silica nanoparticles. J. Phys. Chem. Solids.

[72] Yang C.-C., Wu G. (2009). Study of microporous PVA/PVC composite polymer membrane and it application to MnO2 capacitors. Mater. Chem. Phys..

[73] Kasprzak D., Stepniak I., Galiński M., Stepniak I. (2018). Electrodes and hydrogel electrolytes based on cellulose: Fabrication and characterization as EDLC components. J. Solid State Electrochem..

[74] Fattah N.F.A., Ng H.M., Mahipal Y.K., Numan A., Ramesh S., Ramesh K. (2016). An Approach to Solid-State Electrical Double Layer Capacitors Fabricated with Graphene Oxide-Doped, Ionic Liquid-Based Solid Copolymer Electrolytes. Materials.

[75] Shukur M., Hamsan M., Kadir M. (2019). Investigation of plasticized ionic conductor based on chitosan and ammonium bromide for EDLC application. Mater. Today Proc..

[76] Liew C.-W., Ramesh S., Arof A.K. (2016). Enhanced capacitance of EDLCs (electrical double layer capacitors) based on ionic liquid-added polymer electrolytes. Energy.

[77] Aziz S.B., Hamsan M., Brza M., Kadir M., Muzakir S., Abdulwahid R.T. (2020). Effect of glycerol on EDLC characteristics of chitosan: Methylcellulose polymer blend electrolytes. J. Mater. Res. Technol..

[78] Nadiah N.S., Mahipal Y.K., Numan A., Ramesh S., Ramesh K. (2015). Efficiency of supercapacitor using EC/DMC-based liquid electrolytes with methyl methacrylate (MMA) monomer. Ionics.

[79] Aziz S.B., Hamsan M.H., Karim W.O., Marif A.S., Abdulwahid R.T., Kadir M.F.Z., Brza M.A. (2020). Study of impedance and solid-state double-layer capacitor behavior of proton (H+)-conducting polymer blend electrolyte-based CS:PS polymers. Ionics.

[80] Arof A.K., Kufian M.Z., Shukur M.F., Aziz M.F., Abdelrahman A., Majid S. (2012). Electrical double layer capacitor using poly(methyl methacrylate)-C4BO8Li gel polymer electrolyte and carbonaceous material from shells of mata kucing (Dimocarpus longan) fruit. Electrochim. Acta.

[81] Hamsan M.H., Shukur M.F., Aziz S.B., Yusof Y.M., Kadir M. (2019). Influence of NH 4 Br as an ionic source on the structural/electrical properties of dextran-based biopolymer electrolytes and EDLC application. Bull. Mater. Sci..

[82] Lim C.-S., Teoh K., Liew C.-W., Ramesh S. (2014). Capacitive behavior studies on electrical double layer capacitor using poly (vinyl alcohol)-lithium perchlorate based polymer electrolyte incorporated with TiO2. Mater. Chem. Phys..

[83] Gu H.-B., Kim J.-U., Song H.-W., Park G.-C., Park B.-K. (2000). Electrochemical properties of carbon composite electrode with polymer electrolyte for electric double-layer capacitor. Electrochim. Acta.

[84] Aziz S., Hamsan M., Brza M., Kadir M., Abdulwahid R., Ghareeb H.O., Woo H. (2019). Fabrication of energy storage EDLC device based on CS:PEO polymer blend electrolytes with high Li+ ion transference number. Results Phys..

[85] Kadir M.F.Z., Arof A.K. (2011). Application of PVA-chitosan blend polymer electrolyte membrane in electrical double layer capacitor. Mater. Res. Innov..

[86] Aziz S., Hamsan M.H., Nofal M., Karim W., Brevik I., Brza M.A., Abdulwahid R., Al-Zangana S., Kadir M. (2020). Structural, Impedance and Electrochemical Characteristics of Electrical Double Layer Capacitor Devices Based on Chitosan: Dextran Biopolymer Blend Electrolytes. Polymers.

[87] Aziz S., Hamsan M.H., Karim W., Kadir M., Brza M.A., Abdullah O.G. (2019). High Proton Conducting Polymer Blend Electrolytes Based on Chitosan:Dextran with Constant Specific Capacitance and Energy Density. Biomolecules.

[88] Pandey G.P., Kumar Y., Hashmi S. (2011). Ionic liquid incorporated PEO based polymer electrolyte for electrical double layer capacitors: A comparative study with lithium and magnesium systems. Solid State Ion..

[89] Wang J., Zhao Z., Song S., Ma Q., Liu R. (2018). High Performance Poly(vinyl alcohol)-Based Li-Ion Conducting Gel Polymer Electrolyte Films for Electric Double-Layer Capacitors. Polymers.

[90] Tripathi S.K. (2017). Electrical studies on ionic liquid-based gel polymer electrolyte for its application in EDLCs. Ionics.

[91] Aziz S., Hamsan M.H., Abdullah R.M., Kadir M.F.Z. (2019). A Promising Polymer Blend Electrolytes Based on Chitosan: Methyl Cellulose for EDLC Application with High Specific Capacitance and Energy Density. Molecules.

[92] Liew C.-W., Ramesh S., Arof A. (2016). Investigation of ionic liquid-doped ion conducting polymer electrolytes for carbon-based electric double layer capacitors (EDLCs). Mater. Des..

